# Metabolite 2-aminoadipic acid: implications for metabolic disorders and therapeutic opportunities

**DOI:** 10.3389/fphar.2025.1569020

**Published:** 2025-05-13

**Authors:** Weiyan Shi, Zhiqiang Yang, Pengbin Fu, Yang Yang

**Affiliations:** ^1^ School of Medicine, Xiamen University, Xiamen, Fujian, China; ^2^ Department of Cardiology, School of Medicine, Xiang’an Hospital of Xiamen University, Xiamen University, Xiamen, Fujian, China

**Keywords:** lysine metabolism, 2-aminoadipic acid, diabetes, obesity, atherosclerosis

## Abstract

Previous evidence has indicated that the role of 2-aminoadipic acid (2-AAA), a derivative of lysine catabolism, in mediating specific detrimental effects on glial cells, notably inhibiting astrocyte activation. In addition, intrathecal administration of 2-AAA has demonstrated significant efficacy in relieving mechanical hyperalgesia. With the growing application of metabolomics in biomedical research, substantial evidence now underscores 2-AAA’s pivotal role in regulating glucose and lipid metabolism. As a novel biomarker, 2-AAA is linked to increased susceptibility to diabetes and has emerged as a critical regulator of glucose homeostasis. This review explores recent advancements in understanding 2-AAA’s potential therapeutic applications, particularly in the context of metabolic diseases such as diabetes, obesity, and atherosclerosis. It also addresses existing research gaps and outlines future directions for developing 2-AAA-based therapies.

## 1 Introduction

2-Aminoadipic acid (2-AAA), a key intermediate in lysine metabolism, has emerged as a multifunctional metabolite with significant implications for metabolic and neurological disorders. While traditionally studied for its neurotoxic effects on glial cells (Slawinska et al., 2025), recent advances in metabolomics have repositioned 2-AAA as a dynamic biomarker and modulator of systemic metabolic homeostasis (Fang et al., 2022). Structurally, 2-AAA exists as two isomers: L-α-aminoadipic acid, essential for neurotransmitter regulation and glutamate transport inhibition, and D-α-aminoadipic acid, whose biological roles remain less defined. This duality underscores its complex mechanism of action, spanning from cystine-glutamate antiporter interference (David et al., 2018) to influencing uric acid metabolism in neurological and psychiatric disorders (Fukuwatari, 2020).

The advent of high-resolution metabolomic profiling has revolutionized our understanding of 2-AAA’s clinical relevance. Large-scale cohort studies now link elevated 2-AAA levels to Alzheimer’s disease, schizophrenia, bipolar disorder, subarachnoid hemorrhage, and rheumatoid arthritis (Toledo et al., 2017; Parksepp et al., 2020; Huang et al., 2016; Sokol et al., 2017; Chen et al., 2016). Notably, its role in metabolic dysregulation has gained prominence: 2-AAA is implicated in mitochondrial dysfunction in diabetic cardiomyopathy (Wang et al., 2021) and serves as an independent predictor of type 2 diabetes risk, correlating with insulin resistance and β-cell apoptosis (Lee et al., 2019; Wang et al., 2013). Furthermore, lipidomics-integrated analyses reveal its interplay with acylcarnitines and branched-chain amino acids in obesity-related atherosclerosis, suggesting a broader regulatory network beyond glucose metabolism (Strauss-Kruger et al., 2024).

This review presents the first comprehensive synthesis of 2-AAA’s dual roles in metabolic and neurological pathophysiology, addressing a critical gap in current literature. By integrating recent metabolomic discoveries with decades of biochemical evidence, this work uniquely bridges the historical focus on 2-AAA’s neurotoxic properties with its newly recognized metabolic functions. We pioneer the discussion of 2-AAA as a molecular nexus connecting lysine catabolism to both cerebral and systemic metabolic homeostasis, while proposing novel therapeutic strategies targeting its pathogenic pathways. This cross-disciplinary analysis establishes 2-AAA as a keystone metabolite warranting dual consideration in both neurological and metabolic research paradigms. By bridging fragmented findings from metabolomics, cell biology, and clinical cohorts, this work establishes 2-AAA as a central node in metabolic dysregulation and provides a roadmap for translating mechanistic insights into diagnostic and therapeutic innovations.

## 2 2-AAA metabolic pathways and Enzymatic reactions

**FIGURE 1:**
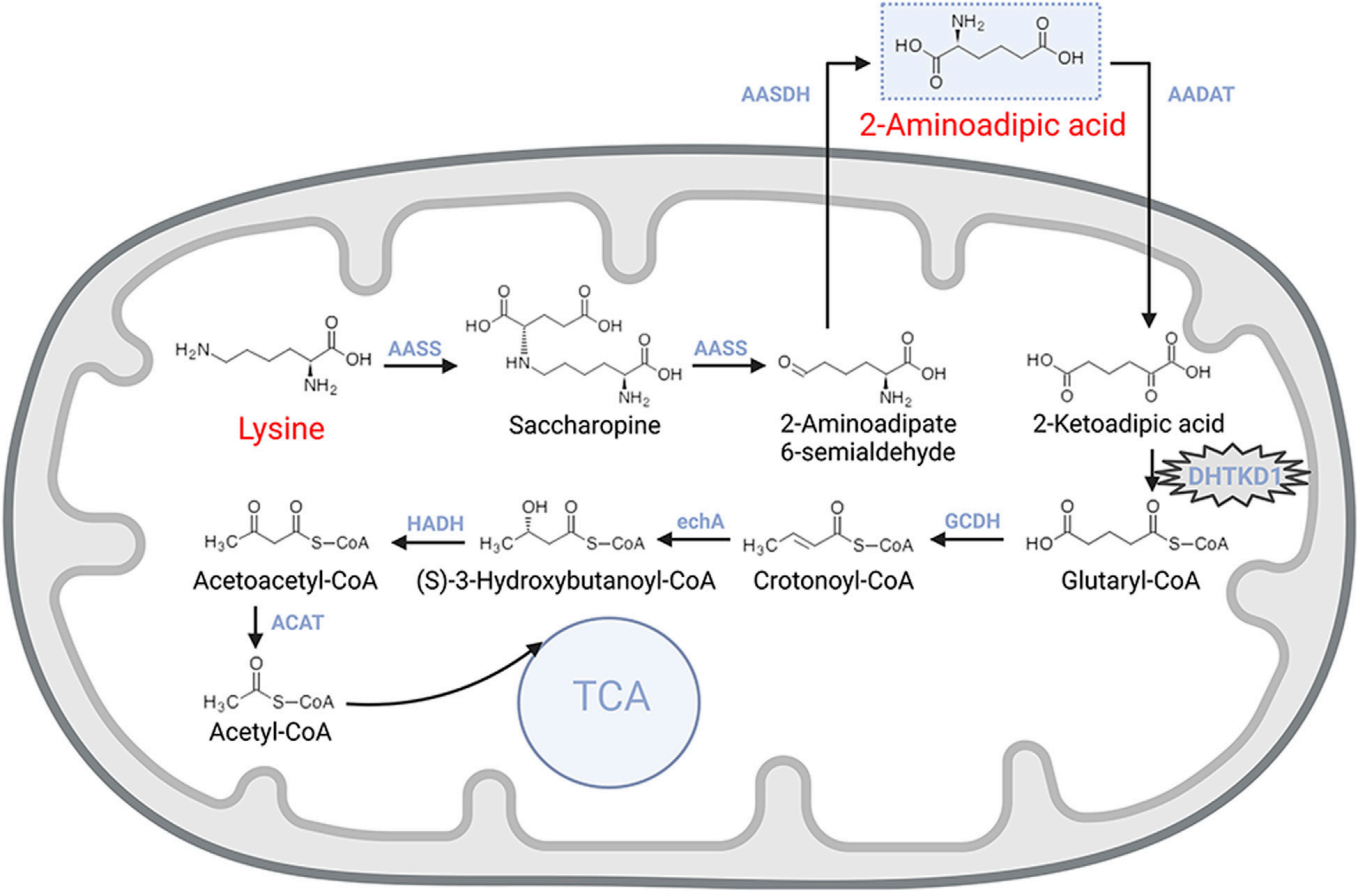
Metabolic pathway of lysine catabolism.

The metabolism of 2-AAA begins with lysine, an essential amino acid actively transported into intestinal mucosal cells and then delivered to the liver via the portal circulation (Matthews, 2020) (Figure 1). In the liver, the enzyme L-lysine-ketoglutarate reductase catalyzes the first step, condensing lysine with α-ketoglutarate to form saccharopine. Saccharopine is subsequently converted into 2-aminohexanedioic semialdehyde by saccharopine dehydrogenase, which is further oxidized to 2-AAA by 2-aminoadipic semialdehyde dehydrogenase. These reactions primarily occur in the mitochondria, with lysine uptake into mitochondria being the rate-limiting step for lysine oxidation (Matthews, 2020; Galili et al., 2001).

The catabolism of 2-AAA follows a pathway similar to that of branched-chain amino acids. The process begins with the transamination of 2-AAA with α-ketoglutarate, yielding 2-ketohexanedioic acid. This intermediate undergoes decarboxylation, catalyzed by a dehydrogenase enzyme, resulting in the production of acetyl-CoA. Further metabolic reactions involving coenzyme A esters continue the breakdown of acetyl-CoA, providing an alternative entry point for the tricarboxylic acid (TCA) cycle, thus integrating 2-AAA metabolism into central energy production pathways (Matthews, 2020).

The degradation of 2-AAA is critically mediated by the dehydrogenase E1 and transketolase domain-containing 1 (*DHTKD1*) genes, which encode proteins that are essential components of the α-ketoglutarate dehydrogenase complex. This complex facilitates the conversion of 2-oxoheptanedioic acid into succinyl-CoA, a key step in the 2-AAA catabolic pathway (Wu et al., 2014; Danhauser et al., 2012). Mutations in the *DHTKD1* gene disrupt this degradation process, leading to metabolic disorders such as 2-aminoadipic aciduria or 2-oxoadipic aciduria, which follow an autosomal recessive inheritance pattern (Danhauser et al., 2012; Stiles et al., 2016).

Furthermore, *DHTKD1* mutations have been implicated in fibular muscular atrophy, highlighting the gene’s broader physiological significance (Xu et al., 2018; Xu et al., 2012). Recent studies indicate that *DHTKD1* plays a crucial role in maintaining mitochondrial morphology and function, with deficiencies leading to impaired mitochondrial integrity. This mitochondrial dysfunction may represent a novel mechanism affecting cardiac metabolism, underscoring the complex role of 2-AAA in regulating both cellular and systemic metabolic pathways (Wang et al., 2021).

## 3 2-AAA as a novel diabetes risk biomarker

**FIGURE 2:**
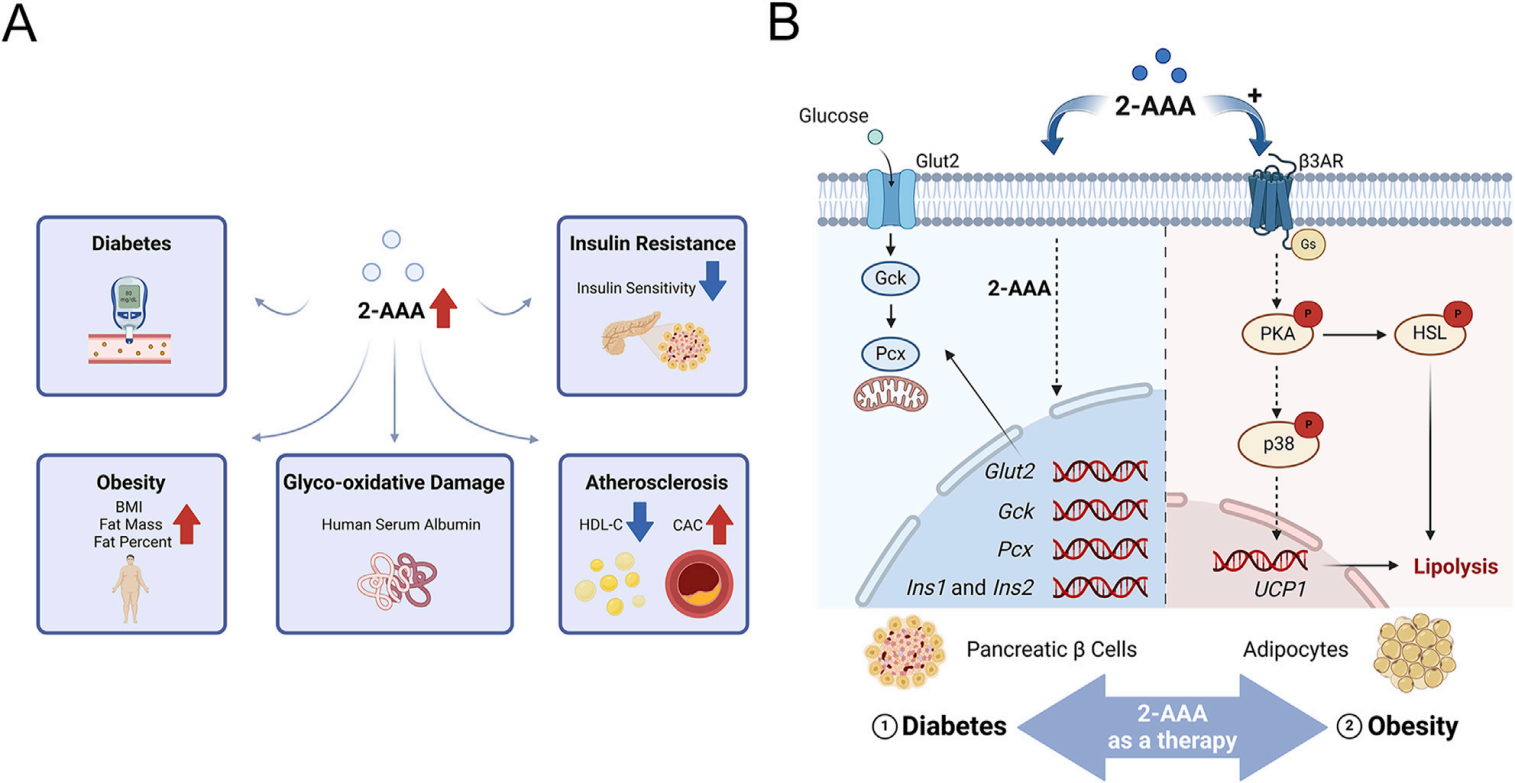
**(A)** 2-Aminoadipic Acid as a Marker of Cardiovascular and Metabolic Diseases.2-AAA is positively correlated with glycoxidative damage, insulin release, and body mass index (BMI), and negatively correlated with high-density lipoprotein cholesterol (HDL-C) and insulin sensitivity. **(B)** Potential Mechanisms of 2-AAA in Insulin Secretion and Lipolysis. This schematic illustrates the proposed pathways by which 2-AAA affects insulin secretion and lipolysis. The primary sites of 2-AAA action are pancreatic β-cells and adipocytes. In pancreatic β-cells, 2-AAA facilitates insulin secretion by regulating the transcription of crucial genes involved in insulin production (*Ins1* and *Ins2*), glucose uptake (*Glut2*), glucose metabolism (*Gck*, *Pcx*), and insulin biosynthesis. Additionally, in adipocytes, 2-AAA overstimulates β3-adrenergic receptor signaling, leading to enhanced lipolysis and thermogenesis.

Diabetes, a group of metabolic disorders of carbohydrate metabolism, is characterized by a condition known as hyperglycemia (American Diabetes Association Professional Practice Committee, 2024). Persistent hyperglycemia can induce target organ damage by increasing the risk of pan-vascular diseases, including microvascular diseases and atherosclerotic macrovascular disorders (Lu et al., 2024). Early identification of individuals at high risk for diabetes, followed by the implementation of effective prevention and intervention strategies, is critical for modern public health management. Data from the Framingham Heart Study, which sought to identify cardiovascular disease (CVD) risk factors, revealed a strong association between 2-AAA levels and diabetes risk. Individuals with elevated 2-AAA levels were found to have a fourfold increase in diabetes risk compared to those with the lowest levels, observed over a 12-year period (Wang et al., 2013). This finding has been further validated in subsequent studies, including in the Chinese population, solidifying the role of 2-AAA as a significant predictor of diabetes (Razquin et al., 2019; Wang et al., 2022) (Figure 2A).

Further insights from *in vitro* experiments indicate that 2-AAA production is triggered only when glucose concentrations exceed normal levels (>7 mM), with its accumulation intensifying alongside glucose concentration increases (Luna et al., 2021). A targeted study under hyperglycemic conditions revealed that human serum albumin significantly promotes the formation of 2-AAA, which serves as a reliable biomarker of glyco-oxidative damage. This effect was observed in the presence of progressively increasing glucose concentrations (Luna et al., 2021). Distinguished from its precursor, allysine, 2-AAA represents a stable byproduct of lysine degradation. Consequently, it has been identified as a critical biomarker for diabetes, offering insights into glucose metabolism disorders and the consequences of elevated blood sugar levels (Luna et al., 2021; Sell et al., 2007). This finding underscores the potential of 2-AAA in facilitating early detection and intervention in diabetes management, thus contributing to the broader understanding and control of this metabolic disorder.

## 4 2-AAA regulation of glucose homeostasis

2-AAA has emerged as a potential regulator of glucose homeostasis, particularly through its influence on pancreatic beta cells, which play a crucial role in controlling blood glucose levels via insulin secretion. Studies, including those conducted by Thomas et al., have shown that exogenous supplementation of 2-AAA significantly reduces fasting blood glucose levels in both chow-fed and Western diet-fed mice (Wang et al., 2013). Further research has demonstrated that 2-AAA promotes insulin secretion in pancreatic beta cell lines, *in vivo* in mice, and in human pancreatic islets (Wang et al., 2013). Notably, exogenous 2-AAA was found to stimulate the transcription of key genes involved in pancreatic insulin production (*Ins1* and *Ins2*), glucose uptake (*Glut2*), glucose metabolism (*Gck*, *Pcx*), and insulin biosynthesis, creating a feedforward loop to sustain β-cell function under metabolic stress (Xu et al., 2018) (Figure 2B). These collective findings underscore the regulatory role of 2-AAA in modulating insulin secretion and maintaining blood glucose levels, highlighting its importance in the broader mechanisms of glucose homeostasis. However, conflicting evidence exists: while animal models demonstrate that 2-AAA supplementation reduces fasting glucose levels, human studies report elevated 2-AAA concentrations in obesity-associated insulin resistance. We hypothesize that chronic hyper-2-AAAemia may reflect β-cell stress, whereas acute 2-AAA administration paradoxically enhances glycemic control by improving insulin sensitivity and β-cell function. Future research must address this dichotomy, underscoring the need for context-specific mechanistic investigations to unravel 2-AAA’s dual roles in metabolic health.

## 5 2-AAA biomarker in insulin resistance

Recent studies have identified 2-AAA as a viable biomarker for insulin resistance, establishing a strong positive correlation between 2-AAA levels, body mass index (BMI), and insulin resistance. This correlation underscores 2-AAA’s potential predictive value for the onset of diabetes (Wang et al., 2013; Ho et al., 2016; Okut et al., 2023). Research involving American Indian adolescents and Korean children with obesity has demonstrated an inverse relationship between plasma 2-AAA levels and insulin sensitivity, with elevated 2-AAA concentrations observed in cellular and mouse models of obesity-related insulin resistance (Short et al., 2019; Lee et al., 2019; Plubell et al., 2018; Aggarwal et al., 2021).

High levels of 2-AAA have been shown to impair insulin signaling in insulin-responsive tissues such as liver, skeletal muscle, and adipocytes, leading to dysregulated gluconeogenesis (Lee et al., 2019). In an obese mouse model characterized by GM-CSF-driven myeloid lineage cell deficiency, insulin sensitivity was maintained despite increased body weight, an observation attributed to the regulatory role of the *Dhtkd1*/2-AAA axis on peripheral insulin sensitivity (Plubell et al., 2018). It is also important to note that modifications in insulin action can influence 2-AAA levels. Studies have highlighted that in obese adults with impaired fasting glucose, plasma 2-AAA concentrations decrease following 3 months of treatment with insulin-sensitizing medications. This reduction is likely due to the capacity of insulin sensitizers to lower oxidative stress and improve insulin sensitivity (Irving et al., 2015).

## 6 Role of 2-AAA in fat metabolism

Adipose tissue has evolved from being considered merely a lipid reservoir to an active participant in modulating insulin sensitivity and diabetes. Specifically, the innervation of adipose tissue in performing the functions of lipolysis and lipogenesis can result in central sympathetic nerve excitation and regulation of distant fat depots and possibly other metabolic tissues to modulate the whole-body glucose homeostasis (Guilherme et al., 2019). When the lipid storage capacity of adipose tissue is exceeded, surplus lipids are redistributed to ectopic sites such as the liver, abdominal region, visceral organs, and skeletal muscles, resulting in ectopic lipid accumulation. This phenomenon leads to dyslipidemia and insulin resistance. Research has revealed significant alterations in 2-AAA levels during the differentiation of human subcutaneous preadipocytes into adipocytes. Specifically, 2-AAA is present in mature adipocytes but absent in precursor cells. These dynamic changes in 2-AAA levels suggest a crucial role in adipogenesis and adipocyte differentiation (Lee et al., 2019). Comparative studies demonstrate a marked increase in plasma 2-AAA levels in obese individuals compared to those of normal weight. This trend is also reflected in the adipose tissue of rodents subjected to a high-fat diet (Lee et al., 2019). Such findings propose a potential involvement of 2-AAA in adipocyte differentiation and its utility as a biomarker for obesity and related metabolic disorders. However, the precise mechanisms underlying these observations require further investigation to fully elucidate 2-AAA’s role in fat metabolism.

Adipose tissue can be classified into two functional types: white adipose tissue (WAT) and brown adipose tissue (BAT). WAT primarily stores energy as triglycerides during periods of caloric surplus and mobilizes these stores as free fatty acids during caloric deficits. WAT is also an essential endocrine organ that secretes a variety of hormones and other factors, collectively known as adipokines, which participate in the regulation of systemic metabolism (Sakers et al., 2022). In contrast, BAT’s primary role is in thermogenesis, utilizing a rich mitochondrial content and the expression of uncoupling protein 1 (UCP1) on the inner mitochondrial membrane to dissipate energy as heat (Scheel et al., 2022; Harb et al., 2023). Interestingly, WAT can undergo a process known as “browning” upon chronic activation of the β3-adrenergic receptor (β3AR). This activation induces WAT to adopt characteristics similar to those of BAT, including enhanced thermogenic function (Cypess et al., 2015; Cero et al., 2021). The activation of brown and beige adipocytes by β3AR agonists significantly reduces fat accumulation, offering a promising strategy for obesity management (Abdul Sater et al., 2022).

Compelling evidence, including studies by Wang et al., demonstrates that exogenous supplementation with 2-AAA significantly reduces body weight and adipocyte size in mice, particularly under a high-fat diet (Xu et al., 2019). These effects are attributed to increased energy expenditure, driven by the upregulation of peroxisome proliferator-activated receptor gamma co-activator-1 alpha (PGC1α) and UCP1, which enhances thermogenesis and promotes lipolysis through elevated expression of hormone-sensitive lipase (Xu et al., 2019). Notably, 2-AAA treatment induces UCP1 expression in WAT, indicating a phenotypic shift towards BAT-like characteristics (Figure 2B). In addition to these thermogenic changes, 2-AAA supplementation improves lipid metabolism by promoting lipogenesis and fatty acid oxidation. This metabolic shift is accompanied by enhanced insulin sensitivity, lower blood glucose levels, and improved glucose tolerance, with pronounced benefits observed under high-fat diet conditions. The administration of 2-AAA mitigates the adverse effects of a high-fat diet, such as increased adipocyte number, excessive triglyceride accumulation in the liver and skeletal muscles, and reduced insulin sensitivity in adipose cells. These benefits are achieved by promoting fat hydrolysis and enhancing insulin action, countering diet-induced obesity (Xu et al., 2019).

Moreover, the phenotypic similarities between *Dhtkd1* knockout mice and those treated with exogenous 2-AAA underscore the role of the *Dhtkd1*/2-AAA axis in regulating lipid metabolism. These findings emphasize the importance of 2-AAA in orchestrating glucose and lipid metabolism, suggesting that increasing 2-AAA levels—either through exogenous supplementation or genetic modulation—could offer a promising therapeutic strategy for obesity and diabetes.

## 7 2-AAA-dyslipidemia mechanistic links

Emerging evidence highlights a significant association between 2-AAA and dyslipidemia, with both human cohort studies and mechanistic investigations suggesting its role in lipid metabolism perturbations. In two independent cohorts—a healthy population (N = 261) and a high-risk group including individuals with treated Human immunodeficiency virus and/or type 2 diabetes (N = 134)—elevated plasma 2-AAA levels were robustly associated with dyslipidemia profiles, specifically lower High-density lipoprotein cholesterol (HDL-C) and higher triglycerides (Desine et al., 2023). These findings were further supported by Mendelian randomization analyses, which identified a suggestive inverse genetic relationship between 2-AAA and HDL-C, implicating variants in lysine degradation pathway genes as potential determinants of this metabolite’s circulating levels (Shi et al., 2022). Mechanistic insights from mouse models reveal nuanced tissue-specific effects: while 2-AAA administration in C57 mice reduced total cholesterol, triglycerides, and Low-density lipoprotein cholesterol (LDL-C) compared to controls, *Dhtkd1* ^*−/−*^ mice exhibited even lower total cholesterol and triglycerides levels alongside elevated HDL-C, suggesting complex regulatory roles in lipid homeostasis that may involve both hepatic and systemic pathways (Xu et al., 2019). Notably, 2-AAA’s interaction with apolipoprotein A-I (apoAI), which is the major HDL protein critical for reverse cholesterol transport, appears particularly relevant, as studies detected 2-AAA-mediated lysine modifications on apoAI that could impair its cholesterol efflux capacity (Peng et al., 2005). This molecular interplay may partially explain the observed HDL dysfunction in high 2-AAA states, though the exact contribution of oxidative modifications versus direct metabolic effects requires further elucidation. Collectively, these findings position 2-AAA as both a biomarker and potential mediator of dyslipidemia, with its dual role in lipid profile alterations and lipoprotein modification underscoring its relevance to cardiometabolic risk.

## 8 2-AAA in atherosclerosis pathogenesis

Atherosclerosis (AS) is a chronic inflammatory pathological process affecting large and medium-sized arteries (Kobiyama and Ley, 2018). Emerging evidence suggests a potential role of 2-AAA in AS progression, though causal mechanisms remain incompletely validated. A recent study demonstrated significantly elevated serum 2-AAA levels in AS patients compared to healthy controls, with moderate positive correlations observed between 2-AAA and inflammatory cytokines, plaque area, and carotid intima-media thickness (Wang et al., 2024). Experimental supplementation of 1% and 2% 2-AAA in drinking water for 13 weeks in AS-prone mice exacerbated aortic plaque formation, as evidenced by increased Oil Red O and HE staining of lesions, suggesting a dose-dependent pro-atherogenic effect (Wang et al., 2024). Mechanistically, 2-AAA may synergize with inflammatory pathways, as its elevation coincided with enhanced expression of NLRP3 inflammasome components in vascular endothelial cells (Wang et al., 2024). However, critical limitations constrain definitive conclusions. The sole reliance on a single preclinical model raises concerns about generalizability, particularly given interspecies differences in lysine metabolism and 2-AAA clearance pathways. Its direct atherogenicity requires validation in human trials and mechanistic studies addressing tissue-specific effects on lipid oxidation, endothelial permeability, and foam cell formation. Given the multifactorial nature of AS, current evidence remains insufficient to confirm causality or exclude confounding interactions with traditional risk factors like dyslipidemia. Multidisciplinary investigations integrating metabolomic profiling, genetic Mendelian randomization, and longitudinal clinical cohorts are warranted to delineate 2-AAA’s role in AS pathogenesis.

## 9 Conclusion

2-AAA, the stable oxidative byproduct of lysine metabolism, has been independently identified as a biomarker for diabetes risk and plays a crucial role in regulating glucose homeostasis *in vivo*. Additionally, 2-AAA is implicated in lipid metabolism and associated with various pathologies, including atherosclerosis and inflammation. The gene *DHTKD1* is integral to the metabolic pathway of 2-AAA, with its expression and functional integrity significantly influencing 2-AAA concentrations and related metabolic phenotypes. Loss of *DHTKD1* is also associated with disruptions in mitochondrial structure and function, underscoring the intricate relationship between 2-AAA metabolism and cellular energy dynamics.

Current clinical investigations of 2-AAA in metabolic diseases are exploratory, focusing on precursor supplementation (e.g., lysine trials: NCT02756117, NCT04417218) and off-target modulation via vigabatrin (NCT04321395). Preclinical studies highlight alternative strategies, such as pharmacological inhibition of DHTKD1, which elevates 2-AAA by blocking its catabolism. These analogues present a promising avenue for reducing the adverse effects of 2-AAA on peripheral nerves, offering a potential therapeutic strategy for managing metabolism-related disorders. As research progresses, understanding the precise mechanisms by which 2-AAA influences metabolic and cardiovascular health, along with its potential therapeutic applications, will become increasingly important.

While emerging evidence implicates 2-AAA in metabolic dysregulation, critical knowledge gaps persist regarding its precise mechanistic contributions to disease pathogenesis. The current understanding remains fragmented, particularly concerning isoform-specific effects and cell-type selective actions in metabolic tissues. Clinical translation faces substantial challenges, including the lack of standardized detection methods for differentiating 2-AAA isomers and insufficient validation in diverse, multi-ethnic cohorts. Significant opportunities exist for future research: 1) Large-scale longitudinal studies to establish causal relationships between 2-AAA fluctuations and metabolic disease progression, 2) Development of isoform-specific pharmacological tools to dissect L/D-2-AAA’s distinct pathophysiological roles, 3) Mechanistic investigations into tissue-specific 2-AAA metabolism, particularly its interplay with mitochondrial function and redox homeostasis. Furthermore, while preclinical DHTKD1 inhibition strategies show promise, their therapeutic potential requires rigorous evaluation of long-term safety and systemic metabolic consequences. Addressing these limitations will be crucial for advancing 2-AAA from a correlative biomarker to an actionable therapeutic target in metabolic medicine.

## Abbreviations

2-AAA, 2-aminoadipic acid; apoAI, apolipoprotein A-I; AS, atherosclerosis; BAT, brown adipose tissue; BMI, body mass index; CAC, coronary artery calcification; CVD, cardiovascular disease; DHTKD1, dehydrogenase E1 and transketolase domain-containing 1; HDL-C, high-density lipoprotein cholesterol; LDL-C, low-density lipoprotein cholesterol; TCA, tricarboxylic acid; UCP1, uncoupling protein 1; WAT, white adipose tissue; β3AR, β3-adrenergic receptor.
